# Amphiphilic Polymer-Modified Uniform CuFeSe_2_ Nanoparticles for CT/MR Dual-Modal Imaging

**DOI:** 10.1155/2020/4891325

**Published:** 2020-12-30

**Authors:** Min Wu, Shaozhi Fu, Jian Shu, Kequan Yu

**Affiliations:** ^1^Department of Radiology, The Affiliated Hospital of Southwest Medical University, Luzhou, Sichuan, China; ^2^Department of Radiology, People's Hospital of Chongqing Yubei District, Yubei District, Chongqing, China; ^3^Department of Oncology, The Affiliated Hospital of Southwest Medical University, Luzhou, Sichuan, China; ^4^Department of Surgery, The First Affiliated Hospital of Chongqing Medical University, Chongqing, China

## Abstract

Recently, magnetic photothermal nanomaterials have attracted much attention in the diagnosis and treatment of cancer. In this study, we developed the ultrasmall magnetic CuFeSe_2_ nanoparticles for CT/MR dual-modal imaging. By controlling the reaction time and condition, CuFeSe_2_ nanoparticles were synthesized by a simple directly aqueous method. After modification with copolymer methoxy polyethylene glycol-polycaprolactone (MPEG-PCL), the obtained MPEG-PCL@CuFeSe_2_ nanoparticles showed excellent water solubility, colloidal stability, and biocompatibility. In addition, they also exhibited superparamagnetism and X-ray's characteristics. For these properties, they will become ideal nanomaterials for CT/MR dual-modal imaging.

## 1. Introduction

In recent years, nanotechnology has been widely used in the field of biomedicine, such as the development of tumor therapeutic drugs and molecular imaging probes [1, 2]. Among them, semiconductor nanomaterials not only have strong fluorescence characteristics but also have fine and uniform particle size, while carbon dots and organic polymer fluorescent nanomaterials have low biological toxicity and relatively stable chemical properties [3, 4]. Many fluorescent nanomaterials, for example, gold-based nanomaterials [5, 6], carbon-based nanomaterials [7], conjugated polymeric nanomaterials [8], and graphene [9–11], are extensively used in photoacoustic imaging and photothermal therapy of tumors, which achieve the purpose of the integration of diagnosis and treatment of tumors.

CuFeSe_2_ is classified as I-III-VI_2_ ternary chalcogenide semiconductor materials, which has interesting optical, electronic, and magnetic properties. Until now, many studies about CuFeSe_2_ mainly focus on the synthesis methods as well as magnetic and optoelectronic properties [12–16]. There are few reports about its application in diagnosis and cancer treatment [17, 18]. Therefore, we will try to explore the potential value of its application in the field of imaging diagnosis.

Currently, the CuFeSe_2_ nanostructures can be prepared by the solvothermal reaction [19] and the high-temperature solid phase reaction [16]. The products which are often synthesized tend to have a nonuniform size and are prone to agglomeration. Therefore, in an effort to overcome their disadvantages of CuFeSe_2_ nanostructures, we attempted to prepare with biodegradable copolymer loaded CuFeSe_2_ nanocrystals to increase the solubility in aqueous media.

MPEG-PCL is an amphiphilic copolymer, and many studies demonstrate that MPEG-PCL copolymer can significantly improve water solubility of hydrophobic drugs and keep better stability [20–22]. Therefore, it can be used to load CuFeSe_2_ nanoparticles for molecular imaging in vivo. First, the properties of PCL, such as crystallinity, tensile strength, and hydrophobicity, can be easily modulated, and thus, the loading capacity for hydrophobic CuFeSe_2_ nanoparticles can be tuned. Second, PEG is nonimmunogenic and highly hydrophilic [23]. Surface coating with PEG can prolong nanoparticle circulation time in vivo, leading to better-enhanced imaging results.

Every imaging modality has its strengths and weaknesses [24]. For instance, X-ray computed tomography (CT) owns its advantages, such as fast acquisition time, large tissue penetration depth, and high spatial resolution, but it has a poor soft-tissue contrast. Magnetic resonance (MR) imaging possesses favorable spatial and soft-tissue resolution, and it can implement multisequence, multiparameter imaging, but its limitation is low sensitivity. Nuclear imaging techniques, including single photon emission computed tomography (SPECT) and positron emission tomography (PET), exhibit high sensitivity and are quantitative, but along with a poor spatial resolution. However, multimodal imaging can improve the accuracy of cancer diagnosis by combining two or more imaging modalities into one system [25, 26]. It overcomes the intrinsic limitations of single modality.

In this study, the ultrasmall magnetic CuFeSe_2_ nanostructures were prepared by a simple direct aqueous method. After modification with methoxy polyethylene glycol-polycaprolactone (MPEG-PCL), the biosafety of obtained MPEG-PCL@CuFeSe_2_ nanoparticles was evaluated. Lastly, the X-ray attenuation property and T_2_MR relaxometry of MPEG-PCL@CuFeSe_2_ NPs in vitro/in vivo were measured to explore the potential application of these NPs as dual-modal CT/MR imaging contrast agents.

## 2. Experimental Section

### 2.1. Materials

Copper (II) chloride dihydrate (CuCl_2_·2H_2_O, ≥99%), ferrous (II) sulfate heptahydrate (FeSO_4_·7H_2_O, ≥99%), selenium powder (Se, ≥99.5%), sodium borohydride (NaBH_4_, 99%), L-cysteine (cys, ≥99%), *ε*-caprolactone (*ε*-CL, Alfa Aesar, USA), poly(ethylene glycol) methyl ether (MPEG, Mn = 2000, Aldrich, USA), and stannous octoate (Sn(Oct)_2_) were bought from Sigma-Aldrich (USA).

### 2.2. Synthesis of CuFeSe_2_ Nanoparticles

For the synthesis of CuFeSe_2_ nanoparticles, 78.96 mg of Se powder was dispersed in 100 mL of Milli-Q water, and then 75.60 mg of NaBH_4_ was added to reduce it at ambient conditions with protection of nitrogen flow for one hour. After Se powder was completely reduced, a colorless solution is obtained. A 10 mL mixture of CuCl_2_ 2H_2_O (85.24 mg), FeSO_4_ 7H_2_O (139.01 mg), and L-cysteine (121.20 mg) was separately prepared, and then the above mixture was added into the selenium precursor solution immediately to form a black solution. The resultant solution was collected after centrifugation with a speed of 3500 rpm for twenty minutes to remove impurities. The purified CuFeSe_2_ solution was stored at 4°C for further characterization and application.

### 2.3. Functionalization of CuFeSe_2_ Nanoparticles

Methoxy poly (ethylene glycol)-poly (*ε*-caprolactone) (MPEG-PCL) used in this study was synthesized by ring-opening polymerization of *ε*-CL on MPEG using Sn(Oct)_2_ as catalyst, according to a previous report [27]. The MPEG-PCL colloidal solution was prepared by liquid rotary evaporation method.

For functionalization of CuFeSe_2_ nanoparticles, the above purified CuFeSe_2_ solution was slowly added into the MPEG-PCL colloidal solution under ultrasonication for 4 hours, and then the MPEG-PCL-modified CuFeSe_2_ nanoparticles were obtained after centrifugation to remove excess and large impurities. The purified MPEG-PCL@CuFeSe_2_ solution was stored at 4°C for future experiments.

### 2.4. Characterization

The hydrodynamic diameters and zeta potentials of prepared CuFeSe_2_ solution and MPEG-PCL@CuFeSe_2_ solution were measured by dynamic light scattering (DLS, NanoBrook 90Plus Zeta, Brookhaven, USA) at 25°C. The size and morphology of prepared CuFeSe_2_ nanoparticles and MPEG-PCL@CuFeSe_2_ nanoparticles were characterized with a transmission electron microscope (TEM, Tecnai G2 F20, USA). The crystallography structures of CuFeSe_2_ nanoparticles and MPEG-PCL@CuFeSe_2_ nanoparticles were characterized by using an X-ray diffractometer equipped with Cu K*α* radiation (*λ* = 0.15406 nm). A scanning rate of 0.1°/s was applied to record the pattern in the 2*θ* range of 10–90°. The T2-weighted images of MPEG-PCL@CuFeSe_2_ at different concentrations were scanned under a 3T clinical MRI scanner at room temperature. After the T2-weighted MR images were acquired, the signal intensity was measured by a manually drawn region-of-interest for each sample.

### 2.5. Cell Culture and Cytotoxicity Assessment

4T1 murine breast cancer cells, A549 human lung adenocarcinoma, and human normal liver cells were cultured in standard cell media supplemented with 10% fetal bovine serum (FBS) and antibiotics (100 U/mL penicillin and 100 *μ*g/mL streptomycin) at 37°C in an atmosphere of 5% CO_2_. All cell culture-related reagents were purchased from HyClone (USA). The cytotoxicity of MPEG-PCL-CuFeSe_2_ NPs was evaluated by the MTT assay. The cells were first seeded into 96-well plates (1 × 10^4^ cells per well) and cultured for 24 h and then added into different concentrations of MPEG-PCL-CuFeSe_2_ and continued to culture for 24 h. After this, 10 *μ*L MTT (5 mg/mL) was added. Four hours later, the supernatant medium was removed, and 150 *μ*L DMSO was added into each well to dissolve the resulting formazan crystals. The absorbance was measured at 490 nm using a spectrophotometric microplate reader (iMark, MA, USA). The cytotoxicity was calculated as the percentage of cell viability.

### 2.6. Animal Model

We acquired female BALB/c mice (6–8 weeks of age, 25–30 g of weight) from Chongqing Tengxin Biotechnology Co. Ltd. (Chongqing, China). To generate the 4T1 tumor murine model, 1 × 10^6^ cells in the 100 *μ*L serum-free RMPI-1640 medium were subcutaneously injected into in the right side thigh root of each mouse. All mice were selected for imaging experiments when their tumors grew to 80 mm^3^.

### 2.7. In Vitro CT/MR Dual-Modality Imaging

Various concentrations of MPEG-PCL@CuFeSe_2_ solution were dispensed in 5.0 mL Eppendorf tubes for CT and MR contrast imaging. The MR imaging for in vitro study was performed on a 3.0 T clinical magnetic resonance (MR) scanner (PHILIPS, Holland). The representative imaging parameters of the T2-weighted images were as follows: repetition time (TR) = 5348 ms, echo time (TE) = 70 ms, slice thickness = 1.5 mm, slice spacing = 0.15 mm, matrix = 256 × 256 pixels, field of view (FOV) = 30 cm × 60 cm × 25 cm, NSA = 4, and flip angle = 10°. The region-of-interest was selected by drawing manually to measure the signal intensity of MPEG-PCL@CuFeSe_2_ solution from the T2-weighted MR images. The CT data were acquired using a clinical CT imaging scanner (GE, USA), and X-ray attenuation values for all samples were finally calculated in Hounsfield units (HU) by averaging over the region-of-interest (ROI). CT imaging parameters were as follows: tube current = 600 mA, tube voltage = 100 kV, and slice thickness = 0.625 mm.

### 2.8. In Vivo CT/MR Dual-Modality Imaging

The 4T1 tumor-bearing mice were acquired before and after intratumorally (i.t.) injected with MPEG-PCL@CuFeSe_2_ (250 *μ*L, 2 mg/mL) and imaged with a 3.0 T clinical magnetic resonance (MR) scanner (PHILIPS, Holland) equipped with a small animal coil. The representative imaging parameters of the T2-weighted images were as follows: repetition time (TR) = 5348 ms, echo time (TE) = 70 ms, slice thickness = 1.5 mm, slice spacing = 0.15 mm, matrix = 256 × 256 pixels, field of view (FOV) = 30 cm × 60 cm × 25 cm, NSA = 4, and flip angle = 10°. The region-of-interest in the tumor area of each mouse was selected by drawing manually to measure the signal intensity of tumors from the T2-weighted MR images. The CT images were acquired before and after intratumorally (i.t.) injected with MPEG-PCL@CuFeSe_2_ (250 *μ*L, 2 mg/mL) on a clinical CT imaging scanner (GE, USA), and the CT imaging parameters were as follows: tube current = 600 mA, tube voltage = 100 kV, and slice thickness = 0.625 mm. The region-of-interest in the tumor area of each mouse was selected by drawing manually to measure the CT value of tumors from the CT images.

### 2.9. In Vivo Toxicity Study

The major organs/tissues were taken from mice after intravenous injection of MPEG-PCL@CuFeSe_2_ (a dose of 20 mg/kg) at 1 day, 3 days, 7 days, and 15 days postinjection, while other mice without injection were used as the control group (four mice per group). Then, the obtained major organs/tissues were fixed in 4% formalin, paraffin-embedded, sectioned, and stained with hematoxylin & eosin (H&E) and then imaged by using a digital microscope to evaluate the histological changes.

## 3. Results and Discussion

### 3.1. Synthesis and Characterizations of MPEG-PCL@CuFeSe_2_ Nanoparticles

**Figure 1:**
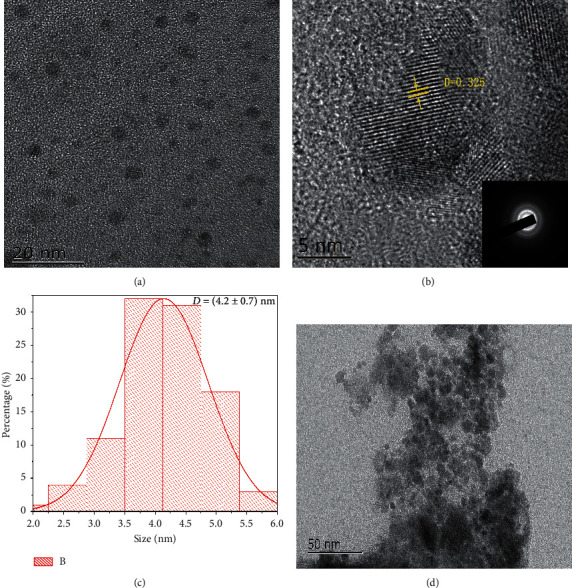
Characterization of as-prepared CuFeSe_2_ and MPEG-PCL@CuFeSe_2_ NPs. (a) TEM image of CuFeSe_2_. (b) HRTEM image with corresponding SAED pattern (inset). (c) The histogram for the measured particle size distribution. (d) TEM image of MPEG-PCL@CuFeSe_2_.

**Figure 2:**
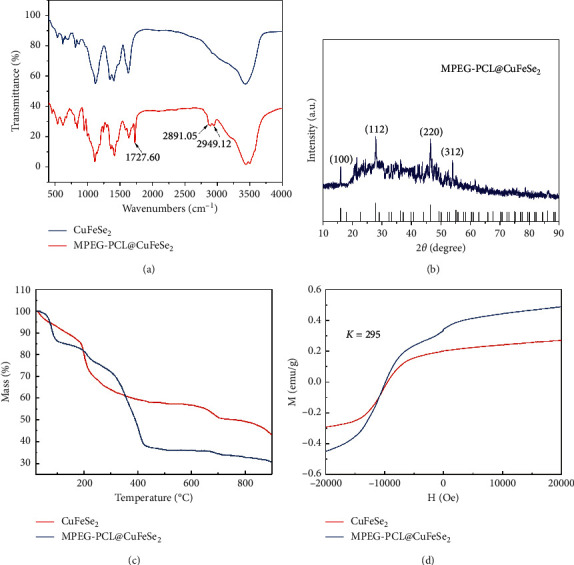
(a) FTIR spectra of CuFeSe_2_ and MPEG-PCL@CuFeSe_2_ NPs. (b) XRD patterns of CuFeSe_2_ prepared with and without MPEG-PCL in comparison with the standard peaks of cubic berzelianite (JCPDS card no. 81-1959). (c) TGA curve of CuFeSe_2_ and MPEG-PCL@CuFeSe_2_ NPs. (d) Magnetization plot of CuFeSe_2_ and MPEG-PCL@CuFeSe_2_ NPs as a function of the applied field at 295K.

In our experiments, MPEG-PCL@CuFeSe_2_ nanoparticles with uniform sizes and morphologies were synthesized by a simple direct aqueous method. The resultant nanoparticles were characterized by transmission electron microscopy (TEM) to determine their size and morphology. Small spherical particles with a size of 4.2 ± 0.7 nm are clearly observed (Figures 1(a), 1(c)). The crystal structure of CuFeSe_2_ nanoparticles was observed by their high-resolution TEM (HR-TEM) image, which clearly shows the lattice fringes with an interplanar spacing of 0.325 nm (Figure 1(b)). The FTIR spectra showed a typical variation peak of C=O at 1727.60 cm^−1^ and typical variation peaks of C-H at 2891.05 cm^−1^ and 2949.12 cm^−1^ in the MPEG-PCL@CuFeSe_2_ nanoparticles, verifying the successful modification of MPEG-PCL (Figure 2(a)).

The X-ray powder diffraction (XRD) results show that both CuFeSe_2_ and MPEG-PCL-CuFeSe_2_ nanoparticles have crystal face peaks at (112) and (220), indicating that the nanoparticles are cubic crystal structures (Figure 2(b)). The content of MPEG-PCL polymer on the surfaces of CuFeSe_2_ nanoparticles was determined by thermogravimetric analysis (TGA) to be approximately 40.0 wt. % (Figure 2(c)), and the TGA results demonstrate the successful coating of CuFeSe_2_ nanoparticles with MPEG-PCL. For the magnetic properties of CuFeSe_2_ and MPEG-PCL@CuFeSe_2_ nanoparticles, the superparamagnetic properties of CuFeSe_2_ and MPEG-PCL@CuFeSe_2_ were illustrated by the absence of a hysteresis loop in the field-dependent magnetization measurement (Figure 2(d)). According to the results from DLS, the hydrodynamic diameters of MPEG-PCL NPs and MPEG-PCL@CuFeSe2 NPs were 25.31 ± 2.07 nm and 120.91 ± 3.44 nm, respectively. MPEG-PCL NPs had a negative surface charge of −21.03 ± 1.53 mV, and MPEG-PCL@CuFeSe2 NPs had a negative surface charge of −10.85 ± 2.59 mV (Table 1).

### 3.2. Toxicity Studies of MPEG-PCL@CuFeSe_2_ Nanoparticles

**Figure 3:**
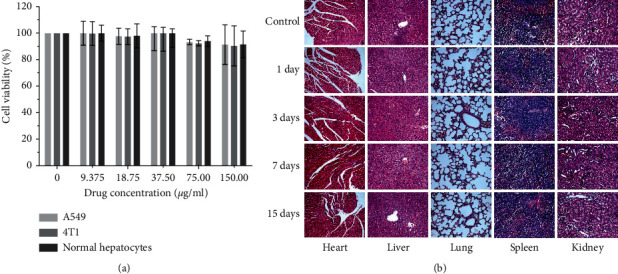
(a) Cytotoxicity studies on 4T1, A549, and human normal liver cells after 24 h incubation with mPEG-PCL@CuFeSe_2_ NPs at different concentrations. (b) Representative H&E stained images of major organs including the heart, liver, spleen, lung, and kidney collected from the tumor-bearing mice at various timepoints after the injection of mPEG-PCL@CuFeSe_2_ NPs, in comparison with those of healthy mice. No obvious organ damage or lesions were observed after treatment.

Good biosecurity is an important criterion for measuring whether nanomaterials can be applied to living organisms. Thus, in vitro MTT assay and in vivo pathological assay were investigated to test the cytotoxicity and biosecurity of MPEG-PCL@CuFeSe_2_ NPs, respectively. The results of MTT assay showed that the viability of the above three kinds of cells still kept above 90% in the concentration range of 0–150 *μ*g/mL for MPEG-PCL@CuFeSe_2_ nanoparticles after cultured 24 h, demonstrating the cytotoxicity is not obvious (Figure 3(a)). In order to test toxicity study of MPEG-PCL@CuFeSe_2_ NPs in vivo, histological assessment of tissues was performed to determine whether MPEG-PCL@-CuFeSe_2_ NPs caused damage to important organs. The above representative organs including heart, liver, spleen, lung, and kidney had no apparent histopathological abnormalities or lesions, compared with those of the control group throughout the entire study. Therefore, the results of in vivo toxicity indicated the good biocompatibility of MPEG-PCL@CuFeSe_2_ NPs (Figure 3(b)).

### 3.3. In Vitro CT/MR Dual-Modality Imaging

**Figure 4:**
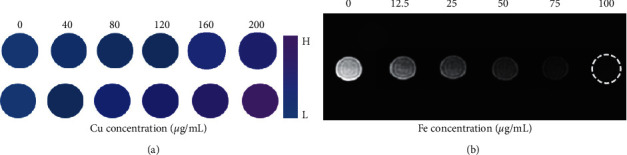
(a) The CT images of mPEG-PCL@CuFeSe_2_ NPs at different concentrations. (b) The T_2_-weighted MR images of mPEG-PCL@-CuFeSe_2_ NPs at different Fe concentrations.

We successfully performed in vitro CT/MR imaging experiment, and it demonstrates the potential of MPEG-PCL@CuFeSe_2_ NPs in CT/MR imaging. As shown in Figure 4(a), from the CT images and Hounsfield unit (HU) values of different concentrations of MPEG-PCL@CuFeSe_2_ NPs in comparison with the clinically used iopromide (Omnipaque, General Electric Pharmaceutical Industry, Shanghai China), with the increasement of the concentration of MPEG-PCL@CuFeSe_2_ NPs and iopromide, the CT signal intensity was gradually enhanced, and at the same concentration, the images are brighter when MPEG-PCL@CuFeSe_2_ NPs are compared with the clinically used iopromide. In addition, the in vitro MRI imaging performance of MPEG-PCL@CuFeSe_2_ NPs was evaluated with a clinically used 3.0 T MRI instrument.  Figure 4(b) shows T_2_-weighted images of MPEG-PCL@CuFeSe_2_ NPs. With the increasing concentration of MPEG-PCL@-CuFeSe_2_ NPs, the MR signal intensity was gradually decreasing, resulting in getting darker images.

### 3.4. In Vivo CT/MR Dual-Modality Imaging

**Figure 5:**
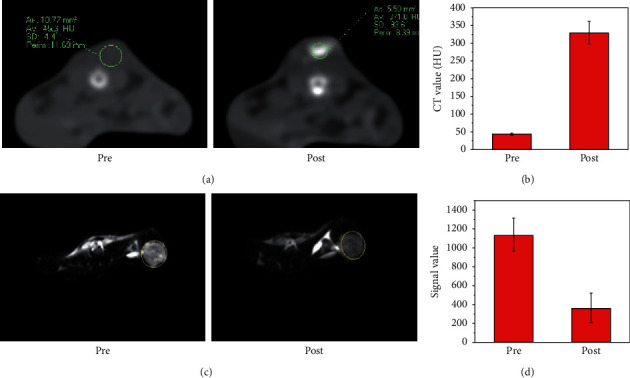
(a, b) CT images and CT values of mice collected pre- and post-intratumoral injection of mPEG-PCL@CuFeSe_2_ NPs. (c, d) T_2_-weighted MR images and signal intensity of mice collected pre- and post-intratumoral injection of mPEG-PCL@CuFeSe_2_ NPs.

Based on the in vitro dual-modal CT/MR contrast performance and good biocompatibility, we performed in vivo CT/MR imaging experiments of mPEG-PCL@CuFeSe_2_ nanoparticles in transplanted mice. In the first place, CT images were acquired before and after intratumoral injection, and the tumor sites showed an enhancement with a higher CT value after administration of contrast agent when compared with those before injection (Figure 5(a)). Similar to CT imaging, MR images were acquired before and after intratumoral injection, and the tumor sites showed an enhancement with a lower T_2_WI MR signal intensity after administration of contrast agent when compared with those before injection (Figure 5(d)). The above results indicate the development of MPEG-PCL@CuFeSe_2_ has the potential to target bimodal CT/MR imaging.

## 4. Conclusions

In summary, the MPEG-PCL copolymer-modified CuFeSe_2_ nanoparticles with favorable biological safety were successfully prepared by an environmentally friendly aqueous route under ambient conditions, and the MPEG-PCL@CuFeSe_2_ nanoparticles perform positive CT/MR contrast effect in vitro/in vivo. These excellent properties enable them to be a promising nanotheranostic agent for in vivo multimodal imaging.

## Figures and Tables

**Table 1:** Hydrodynamic diameters and zeta potentials of NPs.

Nanoparticles	Diameters (d/nm)	PDI	Zeta potential (*φ*/mv)
MPEG-PCL	25.31 ± 2.07	0.263 ± 0.527	−21.03 ± 1.53
MPEG-PCL@CuFeSe2	120.91 ± 3.44	0.183 ± 0.007	−10.85 ± 2.59

PDI: polydispersity index.

## Data Availability

The laboratory experimental data used to support the findings of this study are available from the first author upon request.
